# Syphilis

**Published:** 1846-04

**Authors:** 


*Syphilis.*—M. Ricord observes that the syphilitic poison is eminently alterant and disorganizing, that it produces a manifest alteration in the elements of the blood, and that the essence of the disease is hyposthenic. He reiterates his former denial that any mode of administering mercury after an infectious coition will enable us to secure the immunity for ever of the system from constitutional syphilis. He denies also that mercury produces fever, or causes falling off of the hair; on the contrary, it prevents the latter, and when fever occurs, it behooves the practitioner to look carefully to the lungs and other vital organs. In enumerating the accidents caused by mercury, he cites the case of a man under mercurial treatment, who died of suppuration of the brain, where mercury was obtained after death from the brain by chemical analysis. In a course of lectures on the subject of syphilis by M. Lallemand, now publishing in the "Medical Times," many very important points in reference to the transmission of the disease congenitally are strongly insisted upon, and illustrated by cases. Children are affected with syphilis without the mother ever having exhibited any symptoms, in which case the transmission must be by the father, who at the time of, or for some years previous to the conception of the child, may not have exhibited any evidence of the disease. Cases are detailed of lupus after primary syphilitic and gonorrheal affections; and of the most hideous leprosy, cured by anti-venereals; and others tending to prove, to the contrary of the prevailing opinion, that venereal affections, when neglected or badly treated, are as fatal as formerly.—*Ranking's Abstract.*

